# Exploring the Relation between Emotional Intelligence, Subjective Wellness, and Psychological Distress: A Case Study of University Students in Taiwan

**DOI:** 10.3390/bs11090124

**Published:** 2021-09-10

**Authors:** Inna Reddy Edara

**Affiliations:** Graduate Institute of Educational Leadership & Development, Fu Jen Catholic University, New Taipei City 24205, Taiwan; 065049@mail.fju.edu.tw

**Keywords:** emotions, emotional intelligence, psychological distress, subjective wellness

## Abstract

Given the importance of emotions in human life and the necessity of managing one’s emotions, this research project conducted an 18 week course on emotional management for a group of undergraduate students, investigated the differences in emotional intelligence (EI) levels before and after the course, and assessed EI’s effect on selected subjective wellness and psychological distress variables. The study indicated many significant results. Most importantly, the comparison of the pre-course and post-course EI scores indicated that the EI skills and competencies could be learned and enhanced through formal education. Additionally, there were also significant regression coefficients of pre-course and post-course EI scores on both subjective wellness and psychological distress variables. The significant results endorse that EI knowledge, skills, and competencies could indeed be enhanced through formal education. In particular, the understanding of EI could help the educationists and helping professionals in assessing people’s level of EI, designing relevant courses, and raising the impact of EI on both overall wellness and psychological distress.

## 1. Introduction

Emotions are considered to have a central role in our lives. Although not necessarily always conscious of emotions, humans feel them frequently, and they can be manipulated to achieve certain goals and drive us to perform some impulsive actions. People often want to hide emotions due to various reasons based on individual values and cultural norms. People also usually believe that they need to control their emotions to be acceptable, sound rational, perform better, and live a successful life.

Nevertheless, becoming aware of our emotions and managing them appropriately is all the more difficult as we not only are not conscious of them but do not even understand them well. Incidentally, therefore, people intentionally engage in emotional understanding and management by reading books, attending training programs, and even working with professional helpers. Therefore, given the importance of the emotional part of human life and understanding the necessity of skilled training to understand and manage one’s emotions, this research project tried to describe emotions and elaborate on the skills of emotional management and its relation to subjective wellness and psychological distress among a sample of university students in northern Taiwan.

Emotions simultaneously involve several types of interdependent reactions, such as physiological, biological, cognitive, relational, etc., making it very difficult to have a clear definition of this phenomenon. Nevertheless, scholars try to define and describe emotions in relation to their different fields of research, and thus, most definitions are partial since they only refer to certain aspects of human experience. The following paragraphs briefly describe various theories of emotions, the understanding and development of emotional intelligence (EI), and its measurements and give a short report on EI’s various correlates, particularly the psychological aspects of wellness and distress.

### 1.1. Main Theoretical Trends in Research on Emotions

Research on emotions has been evolving, with many academic fields contributing to the development of numerous theories that attempt to explain the origin, physiology, appraisal, and function of emotions. The main theories include evolutionary theories, physiological theories, cognitive theories, and appraisal theories.

Darwin [1] proposed the first modern theory of emotions, known as evolutionary theory, which recognized the essential role of emotions and their adaptive function in the survival mechanism. Tomkins [2] said that primary emotions are directly triggered by neural activation without any cognitive processing, and they consist in organized physiological, bodily, and facial responses to stimuli. Izard [3] also insisted on the motivational and adaptive significance of emotions, triggered by a specific neural activation that induces in the individual a specific experience that in turn has a specific influence on the individual’s whole behavior, including perception, cognition, and action. Ekman [4] also said that emotions are inherited adaptive functions for mobilizing the organism to deal quickly with important interpersonal encounters based on the adaptive activity from the past. Oatley, Keltner, and Jenkins [5] also believed that the adaptive role of emotions has been shaped during the course of evolution, and they postulated that emotions play a communicative role in the organization of cognitive processes. In sum, it can be said that evolutionary theories highlight the adaptive and communicative functions of emotions, which are inherited and thus do not need cognitive processes.

Contrary to the evolutionary theories that focus on the communicative and adaptive role of emotions, physiological theories consider that physiological activation is the only origin of emotions. James et al. [6] postulated that emotions are genetic reflexes, which are triggered by the perception of physiological changes directly induced by stimuli, which, therefore do not require brain interventions. He said, for example, an individual facing a frightening situation feels the corresponding emotion by noticing or experiencing his or her physiological arousal. In other words, an emotion is a consequence of the perception of physiological arousal. Cannon et al. [7] contradicted James’ [6] assumption that physiological changes are the origins of emotions. According to Cannon, emotion must be felt before the physiological reaction, as the viscera react too slowly. Moreover, physiological changes are quite the same for all emotions; therefore, viscera may not be sensitive enough to differentiate among the emotions. In sum, the physiological theories assume that stimuli cause a physiological activation that triggers emotions.

Cognitive theories assume that neither the inheritance of emotions nor the physiological activation is sufficient to trigger an emotion, because emotion still has to be interpreted, leading to the assumption that cognition is essential in triggering an emotion. The first cognitive theories surfaced in the 1960s (see Arnold [8]; Lazarus [9]; Schacter and Singer [10]; Valins [11]), which endorsed the concept of cognitive aspect as preceding any emotion, which is to say that any specific emotion can only be triggered by the brain reaction through the appraisal of the stimulus or situation. Many cognitive theorists agreed on the importance of physiological activation but also insisted on the need for cognitive interpretation of this activation to identify the felt emotion. Valins [11] went further by saying that physiological activation itself is not necessary, since the belief of activation is sufficient to trigger an emotion.

Along the lines of cognitive theories, Arnold [8] introduced the concept of cognitive appraisal, which is the process of determining the significance of a situation for that individual. This appraisal process triggers an emotion, the corresponding physiological changes, and an action tendency of attraction or repulsion and fight or flight. Lazarus [9] presented a relational, motivational, and cognitive appraisal of emotion, which says that human emotions result from the cognitive appraisal of the interaction between an individual and his/her environment concerning the individual’s motivations. Scherer’s [12] appraisal process considered emotions as a multicomponent process, including a cognitive component, which involves a sequence of stimulus-processing steps, including the evaluation of the novelty of the stimulus, its intrinsic pleasantness, its congruence with goals, its compatibility with norms, and the coping possibilities. On the other hand, Frijda [13] focused on the action tendencies induced by emotions through various steps of cognitive appraisal. According to Frijda, it is the corresponding action tendency that differentiates emotions from each other. Ortony, Clore, and Collins [14] also believed that the origin of emotions is the appraisal of an antecedent situation and that the emotions are valenced reactions to stimuli that differentiate emotions depending on the involved appraisal variables, which may include an appraisal of the desirability of an event, praiseworthiness of an action, and appeal of an object.

Finally, as a summary of the main theoretical trends in research on emotions, it could be said that the study of emotions has been an active field of research in physiology, sociology, and psychology. Such an orientation suggests that emotions are complex phenomena involving various aspects of innate reflexes, physiological activations, cognitive evaluations, social interactions, individual motivation, and subjective feelings. Despite a lack of consensus between these various views, the role of cognition in the triggering and appraisal of emotions and their adaptive function appears to be a widely accepted trend.

### 1.2. Description of Emotions

Currently, there is no consensus on a specific definition of emotions. Various theories that attempt to explain the origin, physiology, cognition, experience, and function of emotions have only fostered ongoing research in this area. Given the explanation of the aforementioned theoretical trends and the continuing intense research on this complex topic, emotions can be described as pleasant or unpleasant states of feeling that are associated with a particular pattern of physiological arousal, cognitive evaluation, and adaptive behavioral tendencies to any given stimulus.

As per this description, emotions are complex to the extent that they are linked to arousal of the nervous system, physiological activity, behavioral tendency, and motivation. As such, emotions involve different components, such as cognitive processes, mental states, contextual appraisals, expressive behaviors, subjective experiences, and cultural labels [15,16]).

### 1.3. History and Models of Emotional Intelligence

Formally linking emotions and intelligence was relatively novel until the 1990s when scholars had begun conceptualizing it as useful for personal, educational, and workplace settings. Both researchers and laypersons alike asked questions, such as: Is emotional intelligence (EI) a new intelligence or just the reframing of existing constructs? Is it an innate ability? Can it be acquired through education and training? What does the existence of an EI mean in everyday life? How does EI affect people’s personality, mental health, relationships, academic achievement, and job performance? [17].

When we track the history, the modern usage of the term emotional intelligence (EI), also known as emotional quotient (EQ) and emotional intelligence quotient (EIQ), seems to have been first mentioned by Beldoch [18] in his paper on “sensitivity to the expression of emotional meaning in three modes of communication” and by Leuner [19] in his paper on “emotional intelligence and emancipation.” Gardner [20] introduced the idea of multiple intelligences, which included some sort of EI. Beasley [21] first used the term EQ. In 1989, Salovey and Mayer [22] put forward another model to describe EI. However, the term EI became popular with the publication of “Emotional Intelligence–Why It Can Matter More Than IQ” by Goleman [23].

When we consider the models of emotional intelligence, it could be said that the outgrowth of emotional intelligence (EI) is a result of two areas of psychological research. The first area is cognition and affect, which involved how cognitive and emotional processes interact to enhance thinking. In other words, it is about understanding how emotions influence how people think, make decisions, and perform different tasks (e.g., Mayer and Bremer [24]; Salovey and Birnbaum [25]). The second area is intelligence itself. It is about the evolution in models of intelligence itself, wherein the scholars had begun considering intelligence as a broader array of mental abilities (e.g., Gardner [20]; Sternberg [26]).

Scholars developed various models to assess EI, such as Petrides and Furnham’s [27] trait model, which focuses on the self-reporting of perceived traits and behavioral dispositions, and Salovey, Mayer, and Caruso’s [28] ability model, which emphasizes the individual’s ability to process emotional information and adapt it to the social environment. Goleman’s [29] model may be considered a mixed model that combines both traits and abilities. In fact, there now seems to be a wide consensus that the ability and trait models to EI are complementary rather than a sign of contradiction or confusion and that both should be included in EI research [30,31]. Recently, research also has focused on the configural model [32], wherein emotional states are attributed to the visual and auditory non-verbal cues, and the neurological model [33], which seeks to characterize the neural mechanisms of EI.

### 1.4. Description of Emotional Intelligence

As there are many models of EI, there are also various descriptions of EI. For instance, Salovey, Mayer, and Caruso [28] defined EI as the ability of individuals in recognizing emotions in themselves and others, discerning between different emotions and feelings, labeling them appropriately, using them to guide thinking and behavior, and adapting them to suit different environments and contexts. This definition is broken into four distinct yet related abilities: perceiving, using, understanding, and managing emotions [34].

Based on the trait model of Petrides and Furnham [27], EI is described as the constellation of individuals’ self-perceptions of their emotional abilities. Goleman [29] defined EI as the drive to performance through an array of five skills and competencies, which include self-awareness, self-regulation, social skills, empathy, and motivation. Goleman said that individuals possess an innate EI that determines their potential for working on their emotional skills and competencies to achieve outstanding performance.

### 1.5. Measurement and Correlates of Emotional Intelligence

The aforementioned multitude of models and descriptions of EI lead to three streams of research measurements based on their underlying assumptions. The three streams are: emotional quotient, emotional ability, and emotional competence [35]. Each of the streams has its corresponding measurement. For example, the stream of emotional quotient (EQ), which focuses on psychological well-being, uses the emotional quotient inventory (EQ-i) to assess EI [36]; the stream of emotional ability, which focuses on emotional reasoning that facilitates thought, uses the Mayer–Salovey–Caruso emotional intelligence test (MSCEIT) to assess EI [37], and the stream of emotional competence, which focuses on behaviors that impact performance, uses the emotional competency inventory (ECI) to assess EI [23,29].

Conceptually, each stream defines the EI construct through its perspective, with some scholars proposing any one of the streams as being more inclusive than others [35]. For instance, Boyatzis [38], who is the leading proponent of the emotional competence stream, is more inclusive in that he distinguishes emotional competence as the behavioral manifestation of innate emotional capacity [35]. Bar-On [36] focuses on the emotional facilitation mechanisms for effective social functioning [35]. Mayer, Salovey, and Caruso [39] are less inclusive, in that they argue that their conceptualization of EI is a set of interrelated emotional reasoning abilities [35]. The present times are witnessing a paradigm shift toward more integrative approaches that recognize that various EI measures reflect distinct strata of a person’s overall EI profile [40,41].

Various correlates of emotional intelligence (EI) have been widely investigated. EI is said to significantly correlate with many variables, including but not limited to social relations, psychological well-being, self-understanding, job performance, leadership, and conflict resolution. It is said to promote positive social functioning and reduce social deviation by helping individuals to detect others’ emotions, enhance communication, and regulate social behavior [17]. For example, a systematic review by Mayer, Roberts, and Barsade [42] found that higher EI is positively correlated with good social interactions and negatively correlated with deviance from social norms. Some studies have shown significant relationships between EI and bullying and cyber victimization [43,44].

Mayer, Roberts, and Barsade [42] said that EI positively correlated with more successful interpersonal relationships and negatively correlated with interpersonal aggression among adults. Bratton, Dodd, and Brown [45] reported that emotionally intelligent people are more likely to achieve self-actualization as they have a better understanding of themselves. Joseph et al. [46] reported a fair amount of predictive power of EI for job performance. Ahmetoglu, Leutner, and Chamorro-Premuzic [47] assessed a possible link between EI and entrepreneurial behaviors and success. Kelly and Kaminskiene [48] and Dabke [49] endorsed the relevance of EI in contexts of business leadership, commercial mediation, and conflict resolution.

In terms of academic performance, EI is postulated to assist in prioritizing thinking and managing emotions [17,50]. The skills and competencies associated with EI help individuals to deal effectively with unpleasant emotions and to promote pleasant emotions to increase personal growth and well-being [17]. A meta-analysis by Schutte et al. [51] and by Martins, Ramalho, and Morin [52] found that EI was associated with better mental and physical health. Mayer, Roberts, and Barsade [42] said that EI also correlated with better psychological well-being and lower levels of depression.

### 1.6. Enhancing Emotional Intelligence

Emotional intelligence (EI) is the knowledge of how emotions function in the self and in others. Current theoretical trends suggest that EI is a combination of dynamic skills that can be learned and enhanced through education, training, and participation [44,53,54]. A few studies that were conducted, indeed, suggested that EI could successfully be enhanced. A study by Chapman [55] indicated that a leadership coaching program specifically designed to enhance leaders’ EI increased their EI as measured by Weisinger’s [56] Boston emotional intelligence questionnaire (BEIQ). Srivastava and Bharamanaikar [57] also suggested that an executive coaching program improved managers’ levels of emotional intelligence competencies. Hess and Bacigalupo [58] reported that organizations and individuals benefitted from the development of EI skills and behaviors that enhanced both individual and group decisions and outcomes. Ebrahimi et al. [59] reported on how enhancing EI impacted reading skills.

Zeidner, Roberts, and Matthews [60] did a critical review on whether emotional intelligence (EI) can be schooled and they found out that most of the intervention programs were not specifically designed to enhance EI. Based on their review, they provided some guidelines for the development, implementation, and evaluation of EI intervention programs, including the need for basing EI programs on a solid conceptual framework, carefully specifying the goals and outcomes, identifying the educational and developmental context for EI interventions, integrating EI programs into the instructional curriculum, and generalizing the domain of EI skills.

In summary, emotions can be described as pleasant or unpleasant states of feeling that are associated with a particular pattern of physiological arousal, cognitive evaluation, and adaptive behavioral tendencies to any given stimulus. Thus, emotions are complex to the extent that they are linked to the arousal of the nervous system, physiological activity, behavioral tendency, and motivation, involving different components, including cognitive processes, contextual appraisals, expressive behaviors, subjective experiences, and cultural labels. Moreover, emotions are considered to have a central role in people’s lives, which can be manipulated to achieve certain goals and perform some impulsive or motivational actions. In addition, individuals usually believe that they need to control their emotions to be acceptable, sound rational, perform better, and live a successful life. In this sense, people use emotional intelligence (EI), which is an ability to perceive emotions in themselves and others, discerning between different emotions and labeling them appropriately, using them to guide thinking and behavior, and adapting them to suit different contexts. As such, EI is a combination of dynamic skills and competencies that can be learned and enhanced through education and training.

### 1.7. Research Purpose and Hypotheses

#### 1.7.1. Purpose

The World Health Organization defines mental health as a state of well-being in which individuals realize their own abilities, can cope with the normal stresses of life, work productively, and are able to make a contribution to their community [61]. As per this description, there are two distinct dimensions in mental health: a positive dimension, corresponding to psychological well-being, and a negative dimension, corresponding to psychological distress and mental disorders.

Therefore, the evaluation of student mental health should investigate both dimensions by taking into account students’ psychological distress as well as their psychological well-being. However, most studies on student mental health have examined psychological distress only, typically assessed in terms of depression, anxiety, and stress. In general, university students report high psychological distress scores [62,63]. A study investigating the links between psychological distress and health behaviors found that depressive symptoms correlated in students with skipping breakfast, low sleep quality, inadequate physical activity, and short or long sleep hours [64]. A qualitative study identified various sources of stress, including practicum, theoretical training, personal life, and social life [65]. 

Compared to research on students’ psychological distress, only a few studies examined their psychological well-being. One qualitative study found that most students had a good quality of life and were satisfied with their health and how they lived [63]. Dyson and Renk [66] studied freshmen adaptation to university life and coping. 

In summary, much research on student mental health has focused on psychological distress, and only a few studies investigated psychological well-being. However, an inclusive approach should examine both positive and negative dimensions, thereby including both coping and risk factors. Additionally, studies have mostly focused on professional students, such as medical and nursing students. It is necessary to study students of other disciplines as well. Therefore, the main objective of this research was to study the mental health status of bachelor’s degree students training in different disciplines by exploring both psychological distress and well-being and their relation to emotional development and management. 

#### 1.7.2. Hypotheses

Based on the description of emotions in general and emotional intelligence (EI) and there being significant correlates in particular, this research attempted to investigate the effect of EI on selected subjective wellness and psychological distress variables among a group of college students in a formal educational setting (Study 1). In addition, believing that EI skills and competencies can be enhanced through formal education and training, this research study also conducted an 18 week course on emotional management for a group of undergraduate students; at the end of which, it was investigated for the differences in EI levels and also the EI’s effect on selected subjective wellness and psychological distress variables (Study 2).

Specifically, this research project tested the following hypotheses:

*Study 1*

1. There would be acceptable reliability coefficients of the scales and significant correlations among the study variables. For example, there would be a significant positive correlation between two wellness variables of optimism and hope, suggesting that both the variables mutually influence each other in the same direction.
2. There would be significant effects of emotional intelligence on the participants’ subjective wellness and psychological distress dimensions. For instance, higher levels of EI would predict lower levels of the psychological distress variable of depression.

*Study 2*

1. There would be acceptable reliability coefficients of the scales and significant correlations among the study variables. For example, there would be a significant negative correlation between the wellness variable of hope and the psychological distress variable of depression, suggesting that both the variables mutually influence each other in the opposite direction.
2. Due to the formal academic course in emotional management and implementation of the appropriate interventions throughout the 18 week academic semester, there would be significant increases from the pre-course to post-course scores on the emotional intelligence of the participants.
3. There would be significant effects of the post-course emotional intelligence levels on the participants’ subjective wellness and psychological distress dimensions. In addition, the significant effects would be different from those of the pre-course regression analyses, suggesting the relevance of a formal course in emotional management to enhance EI.

## 2. Methods

### 2.1. Study 1

#### 2.1.1. Procedure and Participants

The research participants were students of a prominent university in the northern part of Taiwan. After reading and signing the informed consent form during a class hour, a total of 202 voluntary participants from five departments completed the study questionnaire. The five academic departments included educational leadership, Chinese literature, clinical psychology, life-science, and English literature. Sample demographics are given in Table 1. As reported in Table 1, there were more female students (69.8%) than male students (29.7%) with their ages ranging from 18 to 25. The participants were almost equally distributed among the five academic departments.

#### 2.1.2. Measures

Emotional Intelligence. The emotional intelligence (EI) was measured by the Boston EI Questionnaire (EIQ), which was developed by Clarke [67] based on the work of Weisinger [56] who described EI as comprising self-awareness, managing emotions, self-motivation, relating to others, and emotional mentoring. This scale was translated into Chinese and used with Chinese-speaking samples, which had a reliability of 0.91 [68]. The Boston EIQ scale consists of 25 items, measured on a continuous scale from 1 to 10, with higher scores suggesting higher EI. As reported in Table 2, the reliability for the total scale was 0.94 in this study. Sample items included, “Can you tell when your mood is changing” and “How well can you concentrate when you are feeling anxious?”

Optimism. Optimism was measured by the life orientation test-revised (LOT-R), developed by Scheier, Carver, and Bridges [69]. The LOT-R scale was translated into Chinese and used with Chinese-speaking samples, which had a reliability in the range of 0.87 [70,71]. The LOT-R is a 6-item self-report scale, with 3 positively worded and 3 negatively worded items, measured on a 5-point Likert scale, ranging from 1 (strongly disagree) to 5 (strongly agree). After reverse-coding the negatively worded items, the six items were summed to produce an overall score, with higher scores representing more optimism. As reported in Table 2, the alpha coefficient for LOT-R in this study was 0.80. Sample items included, “In uncertain times, I usually expect the best” and “I rarely count on good things happening to me (reversed).”

Satisfaction with Life (SWL). SWL is a 5-item instrument designed to measure overall life satisfaction based on individual perceptions of subjective well-being [72]. This scale was translated into Chinese and widely used in many studies with good reliability values above 0.85 [73]. The SWL uses 7-point Likert-type responses, ranging from 1 (strongly disagree) to 7 (strongly agree). The five items are summed for an overall score, with high scores indicating that an individual is generally more satisfied with his or her life. As reported in Table 2, the reliability coefficient for SWL in this study was 0.82. Sample items included, “In most ways, my life is close to my ideal” and “If I could live my life over, I would change almost nothing.”

Hope. Hope was assessed by Snyder et al.’s [74] adult hope scale (AHS). The AHS scale is comprised of 12 items, using Likert-type responses, ranging from 1 (strongly disagree) to 5 (strongly agree). The AHS was translated into Chinese language and measured a good internal consistency of 0.80 [75]. This scale was designed to measure the reciprocal interaction between goal-directed thoughts (agency) and goal-directed actions (pathways). The two subscales (agency and pathways) use four items each. Moreover, four additional items are used as distracters. The two subscales were summed to create an overall hope score. High scores on the overall hope scale indicate individuals are more hopeful, more motivated to achieve their goals, and more capable of designing means to achieve their goals. As reported in Table 2, the reliability alpha for the hope scale in this study was 0.74. Sample items included, “I energetically pursue my goals” and “There are lots of ways around my problem.”

Psychological Distress. This was assessed by the brief symptom rating scale (BSRS-5), developed by Lee et al. [76] to identify psychiatric morbidity or psychological distress in both medical practice and the general community. The items are measured on a Likert scale, ranging from 0 (not at all) to 4 (very severe). As reported in Table 2, the reliability coefficient for this scale in this study was 0.82. Sample items included, “Have problems with sleeping” and “Feel anxious and low”.

Depression. The levels of depression were measured by the Taiwanese Depression Questionnaire, developed by Lee et al. [77]. This scale consisted of 18 statements, measured on a Likert scale, values ranging from 0 (less than one day per week) to 3 (5 to 7 days per week). As reported in Table 2, the reliability coefficient for this scale was 0.92 in this study. Sample items included, “I felt blue and depressed” and “I tended to look at the dark side of everything”.

Demographics: The demographic form included participant’s gender, age, and academic department.

#### 2.1.3. Statistical Analyses

Collected data were analyzed using SPSS version 20.0 (IBM Corporation: Armonk, NY, USA) on loan from the researcher’s university. Descriptive statistics such as mean, standard deviation, and frequency were analyzed to describe the data distribution. Reliability coefficients were investigated to assess the scales’ internal consistencies and reliabilities. Pearson’s correlations were used to assess and interpret the correlations between the study variables. Finally, hierarchical multiple regression analyses were conducted to investigate the impact of emotional intelligence on the wellness and distress variables of the participants.

### 2.2. Study 2

#### 2.2.1. Procedure and Participants

The research participants were 46 students from educational leadership department at a prominent university in northern Taiwan, who voluntarily enrolled in a 2-credit 1-semester (18-week) course on emotional management. Of the 46 participants, 20 (43.5%) were male, and 26 (56.5%) were female, with their ages ranging from 18 to 21.

The students attended two-hour classes each week and met with the course instructor regularly to discuss various topics and interventions related to emotional management. Some of the major topics, interventions, and assignments covered in this course are indicated in Table 3.

As it can be seen from Table 3, the course topics included that of concepts, physiology, cognition, personality, family, society and culture, the influence of media and technology, and metaphors. The interventions and assignments included movies and analysis, discussion of prominent figures’ EI, completing the relevant questionnaire, in-vivo exercises, weekly journaling, and semester integration paper. The course evaluation at the end of the semester by the students about the course and its contents, measured on a continuous Likert-scale, with values ranging from 1 (do not like or not satisfied at all) to 10 (very much like it or very satisfied), indicated the mean values in the range of 6.46–9.00. Grand mean was 7.37, suggesting that the students overall favored the course and felt highly satisfied.

#### 2.2.2. Measures

Students in Study 2 completed the same package of the questionnaire that was used in Study 1, both at the beginning of the semester and at the end of the semester, to assess the scores at pre-course and post-course levels for the subsequent comparisons and analyses. Reliability coefficients are presented in Table 4. As it can be noticed in Table 4, all the measures in both pre-course and post-course analyses had acceptable reliability coefficients with values ranging from 0.74 to 0.96 in pre-course analysis and from 0.76 to 0.96 in post-course analysis.

#### 2.2.3. Statistical Analyses

The SPSS version 20.0 on loan from the researcher’s university was used to analyze the collected data. Descriptive statistics such as mean, standard deviation, and frequency were analyzed to describe the data distribution. Reliability coefficients were investigated to assess the scales’ internal consistencies and reliabilities. Pearson’s correlations were used to assess and interpret the significant bivariate correlations. Hierarchical multiple regression analyses were conducted to investigate the impact of emotional intelligence on the components of subjective wellness and psychological distress. Finally, paired-samples *t*-tests were accomplished to evaluate the differences in scores of the major study variables before and after the course on emotional management.

## 3. Results

### 3.1. Study 1

#### 3.1.1. Intercorrelations

As given in Table 2, all the intercorrelations among the study variables were significant, except for the correlation between hope and psychological distress. The correlational values ranged from 0.15 to 0.74, suggesting the range of small to high correlation coefficients [78]. There was a strong positive correlation between psychological distress and depression, *r* = 0.74, *p* < 0.01, with higher levels of distress associated with higher levels of depression. There was also a strong negative correlation between optimism and depression, *r* = −0.52, *p* < 0.01, and between satisfaction with life and depression, *r* = −0.52, *p* < 0.01, suggesting that higher levels of depression were associated with lower levels of optimism and satisfaction with life. As expected, there was a strong positive correlation between optimism and satisfaction with life, *r* = 0.51, *p* < 0.01, with higher scores on optimism associated with higher scores on satisfaction with life and vice versa.

#### 3.1.2. Regression Analyses

Hierarchical multiple regression analyses were conducted to assess the ability of emotional intelligence (EI) to predict the levels of optimism, satisfaction with life, hope, psychological distress, and depression, after controlling for the demographics. The results are presented in Table 5.

As indicated in Table 5, EI significantly predicted all the outcome variables. Examining the effect sizes, it could be seen that that the participants’ EI had the highest effect on the subjective wellness variable of hope, *t* (201) = 6.80, *p* < 0.01, *β* = 0.43, followed by optimism, *t* (201) = 3.85, *p* < 0.01, *β* = 0.26, and satisfaction with life, *t* (201) = 3.85, *p* < 0.01, *β* = 0.26. These results suggested that the participants who had higher EI experienced higher levels of hope, optimism, and satisfaction with life. Regarding the distress variables, EI had a higher effect on depression, *t* (201) = −4.15, *p* < 0.001, *β* = −0.28, than on psychological distress, *t* (201) = −2.08, *p* < 0.05, *β* = −0.14. These significant results suggested that those university students of this study who had lower EI levels experienced higher levels of depression symptoms and psychological distress.

### 3.2. Study 2

#### 3.2.1. Intercorrelations

The intercorrelations for the major study variables in both pre-course and post-course analyses are given in Table 4. Most of the intercorrelations were significant, with the values ranging from 0.15 to 0.48 in the pre-course analysis and from 0.13 to 0.56 in the post-course analysis, suggesting the range of small to high correlation coefficients [78].

In pre-course analysis, there was a strong positive correlation between psychological distress and depression, *r* = 0.74, *p* < 0.01, suggesting that higher levels of distress were associated with higher levels of depression, whereas this association was reduced in post-course analysis, *r* = 0.48, *p* < 0.01. There was also a strong positive correlation between emotional intelligence and hope in pre-course analysis, *r* = 0.61, *p* < 0.01, indicating that the participants who had better skills in managing emotions had higher levels of hope, and vice versa. In post-course analysis, the strongest correlation was between hope and depression, *r* = −0.54, *p* < 0.01, suggesting that higher levels of depression were associated with lower levels of hope. As expected, there was a strong negative correlation between emotional intelligence and depression, *r* = −0.43, *p* < 0.01, with lower scores on emotional intelligence associated with more depressive symptoms.

#### 3.2.2. Differences in Pre-Course and Post-Course Scores

**Figure 1:**
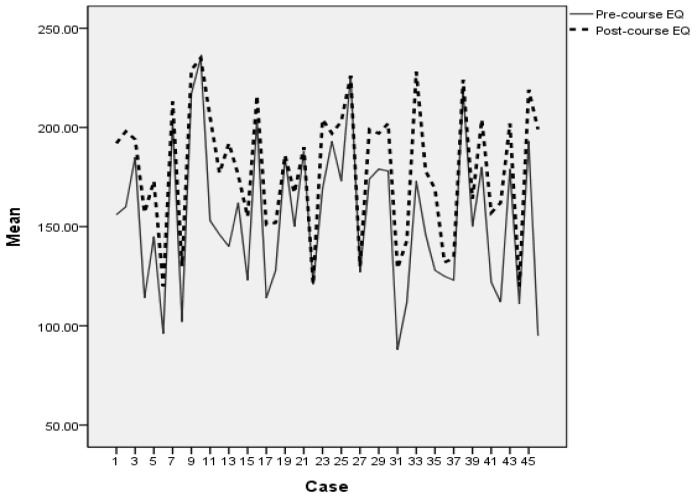
Pre-course and post-course emotional intelligence scores.

A group of paired-samples *t*-tests was conducted to evaluate the impact of emotional management course on the levels of participants’ EI, optimism, satisfaction with life, hope, psychological distress, and depression. As indicated in Table 6, there was a statistically significant difference for all the variables from pre-course to post-course. For example, as reported in Table 6 and Figure 1, there was a statistically significant increase in overall EI scores, *t* (45) = 8.67, *p* < 0.001, from pre-course (M = 154.39, SD = 37.94) to post-course (M = 179.37, SD = 33.17). The mean increase in EI scores was 24.98 with a 95% confidence interval ranging from −30.77 to −19.17. The eta squared statistic, calculated using the formula ([79], p. 247), *η*^2^ = *t*^2^/*t*^2^ + (*N*−1), indicated a large effect size (*η^2^* = 0.63).

As it can be noticed in Figure 1, most of the participants had a significant increase in their EI levels after attending the course on emotional management. For a few participants, such as numbers 21, 27, and 43, there was no significant difference in their EI levels even after taking the course on emotional management.

Among the other variables, depression had a significant decrease from pre-course (M = 31.09, SD = 8.27) to post-course (M = 16.54, SD = 4.39), *t* (45) = 19.40, *p* < 0.001, *η^2^* = 0.89. The mean decrease in depression scores was 14.54 with a 95% confidence interval ranging from 13.03 to 16.05. Satisfaction with life had a significant increase from pre-course (M = 19.52, SD = 6.23) to post-course (M = 29.89, SD = 2.95), *t* (45) = 16.97, *p* < 0.001, *η^2^* = 0.86. The mean increase in satisfaction with life scores was 10.37 with 95% confidence intervals falling between −11.03 and −9.13. Overall, the significant increase in EI and wellness variables and decrease in distress variables suggested that the structured course on emotional management was helpful to the participants.

#### 3.2.3. Regression Analyses

This research also conducted regression analyses to test, respectively, the effect of pre-course and post-course EI levels on both pre-course and post-course scores of optimism, hope, satisfaction with life, psychological distress, and depression. The results are presented in Table 7.

As indicated in Table 7, in the pre-course analyses, only the regression model of EI on hope was significant, *F* (1, 45) = 26.08, *p* < 0.01, *R*^2^ = 0.37, and it was not significant for the rest of the outcome variables. In other words, the levels of EI before taking the course on emotional management had a significant contribution to the subjects’ hope, *t* (45) = 5.11, *p* < 0. 001, *β* = 0.61.

In the post-course analyses, as reported in Table 7, the models for satisfaction with life, *F* (1, 45) = 6.80, *p* < 0.05, *R*^2^ = 0.13; hope, *F* (1, 45) = 10.82, *p* < 0.01, *R*^2^ = 0.20, and depression, *F* (1, 45) = 9.96, *p* < 0.01, *R*^2^ = 0.19, were significant. In other words, the increase in levels of EI after taking the course on emotional management had a significant contribution in increasing the subjects’ hope, *t* (45) = 3.29, *p* < 0.01, *β* = 0.44, and satisfaction with life, *t* (45) = 2.61, *p* < 0.05, *β* = 0.37, and in decreasing the levels of depression, *t* (45) = −3.16, *p* < 0.01, *β* = −0.43.

## 4. Discussion

Briefly stated, emotional intelligence (EI) is knowledge and a set of skills of how emotions function in self and others in a given context. Current theoretical trends suggest that EI is a combination of dynamic skills and competencies that can be learned and enhanced through education, training, and participation. It is also believed that EI has a multitude of significant correlates. Given these assumptions, this research project (Study 1) attempted to investigate the correlations and effects of EI on selected subjective wellness and psychological distress variables among a sample of university students in northern Taiwan. Further, believing that EI skills and competencies can be enhanced through formal education and training, this research study (Study 2) also conducted an 18-week academic and practicum course on emotional management for a class of undergrad students to investigate the impact of a formal course on EI levels and also to assess the EI’s effect on selected subjective wellness and psychological distress variables. Appropriate statistical analyses suggested various significant results in both Study 1 and Study 2, which are discussed in the following paragraphs.

### 4.1. Mutual Correlations

There was a strong positive correlation between psychological distress and depression, in both Study 1 and Study 2, indicating higher levels of distress associated with higher levels of depression. Psychological distress as measured by the brief symptom rating scale (BSRS-5) is generally used to identify minor mental disorders associated with depression and anxiety in non-psychiatric clinical settings [76]. Psychological distress differs from organic mental disorders, say depression, in the sense that distress is a reactive symptom affected by external stress, and it refers to a broader affective and emotional experience than a depressive disorder [80]. Psychological distress, depending on its duration and intensity, could lead to depression [81].

The positive variables of optimism, satisfaction with life, and hope also significantly and inversely correlated with depression, in both Study 1 and Study 2 (pre-course), suggesting that the higher levels of subjective wellness relate to lower levels of depression. Studies have reported that positive psychological characteristics, such as optimism and hope, may act as protective factors for depression distinct from the absence of negative characteristics [82]. According to positive psychology, the constructs of optimism, hope, and satisfaction with life are cognitive components of the subjective wellness spectrum. Cognitive theories suggest that negative automatic thoughts play a key role in the onset of depression. Therefore, it is natural that positive cognitive components act as a buffer against the negative thoughts and feelings of depression. 

As expected, there was a strong positive correlation between hope, optimism, and satisfaction with life, in both Study 1 and Study 2, suggesting that if people are more hopeful and optimistic, then they are more satisfied in their lives. Both optimism and hope are said to involve goal-based cognitive processes in achieving a valued perceived outcome [83]. Satisfaction with life is described as the cognitive evaluation of one’s life [72]. Therefore, and rightly so, the cognitive processes of hope and optimism positively evaluate the component of life satisfaction. 

In Study 1, emotional intelligence (EI) significantly correlated with all the wellness and distress variables. In Study 2 (pre-course), EI significantly correlated only with hope, whereas in Study 2 (post-course), it significantly positively correlated with satisfaction with life and hope and negatively correlated with depression. Studies have suggested that highly emotionally intelligent individuals are likely to experience higher levels of well-being than individuals with low emotional intelligence, whereas individuals with lower levels of EI experience emotional deficit and are prone to dealing with distress and depressive symptoms [84]. In other words, EI intuitively offers a window into mental health or illness, since EI is described as the ability of individuals to understand their emotional states, which are considered as important indicators of healthy or unhealthy mental functioning [85].

### 4.2. Role of Emotional Management Course in EI and Other Variables

Emotional intelligence (EI) is a knowledge and awareness of emotions and how they function in self and others. Current theoretical models and research trends suggest that EI is a combination of dynamic skills and competencies that can be learned and enhanced through education and training [44,53,54].

As the results of paired-samples *t*-tests reported (see Table 6), a structured emotional management course made a significant difference in the expected direction on the levels of participants’ EI levels from pre-course to post-course analysis. The mean increase in EI scores was very large. In addition, as indicated in Figure 1, most of the participants had a significant increase in their EI levels after attending the course on emotional management. 

As Zeidner et al. [60] recommended, it was possible to enhance the EI scores of this study participants because the structured course on EI enhancement was integrated into the instructional curriculum of an undergrad program. It was also based on a clear conceptual framework that included areas of physiology, emotions, cognition, motivation, personality, family, culture and society, and media and technology. Such a framework conforms to the model that emotions involve different components, such as cognitive processes, contextual appraisals, expressive behaviors, subjective experiences, and sociocultural labels [15].

Zeidner, et al. [60] also recommended to carefully specify the goals and outcomes of EI enhancement programs, identify the educational and developmental context for EI interventions, and generalize the domain of EI skills. In the structured course on emotional management, these recommendations were implemented through group discussions and oral reports, in-class group activities and exercises, weekly journal writing, and a final integration paper. As the results have indicated (see Table 2), all these content areas and skill domains must have helped the participants enhance their EI levels.

Structured emotional management course also made a significant difference in the expected direction on the levels of participants’ optimism, satisfaction with life, hope, psychological distress, and depression from pre-course to post-course analysis. For example, depression had a significant decrease from pre-course to post-course, with a mean decrease of 14.54, and psychological distress had a mean decrease of 5.08. The mean increase in satisfaction with life scores was 10.37; it was a 7.74 point increase for hope and a 6.74 point increase for optimism (see Table 6). These results suggest that a well-designed course on the enhancement of EI not only enhances EI but also simultaneously changes other aspects of the participants’ lives in the desired directions. Overall, the significant increase in EI and wellness variables and decrease in distress variables suggested that the structured course on emotional management was helpful to the participants. 

### 4.3. Impact of EI on Wellness and Distress

In Study 1, hierarchical multiple regression analyses were conducted to assess the ability of emotional intelligence (EI) to predict the participants’ levels of optimism, satisfaction with life, hope, psychological distress, and depression. As reported in Table 5, EI significantly predicted all the outcome variables, indicating that the participants who had higher levels of EI experienced more hope, optimism, and satisfaction with life. Regarding the distress variables, the significant results suggested that the participants who had lower EI levels experienced higher levels of depression symptoms and psychological distress.

This research also conducted regression analyses (Study 2) to test respectively the effect of pre-course and post-course EI levels on both pre-course and post-course scores of the wellness variables of optimism, hope, satisfaction with life, and the distress variables of psychological distress and depression. As reported in Table 7, in the pre-course analyses, only the regression model of EI on hope was significant. In the post-course analyses, the models for satisfaction with life, hope, and depression were significant. In other words, the increase in levels of EI after taking the course on emotional management had a significant contribution to increasing the participants’ hope and satisfaction with life and to decreasing the levels of depression.

The variations in regression coefficients in Study 1 and Study 2 could be attributed to the differences in sample size. Study 1 consisted of a larger sample in comparison to Study 2. Moreover, only Study 2 participants went through a course on emotional management in enhancing EI. Whether in Study 1 or Study 2, it is clear from the analyses that EI, both before the training and after the enhancement course, impacts the wellness and distress of the participants in the expected directions. 

In general, these results replicate the results from other research studies (see Acosta and Clavero [86]; Extremera and Fernandez-Berrocal [87]; Liu, Wang, and Lu [88]). To be precise, this study’s results suggest that as the emotional intelligence (EI) increases, individuals’ subjective wellness increases and psychological distress decreases. That is, the ability to be optimistic, hopeful, and satisfied in life increases with the enhancement of EI. Inversely, with deficits in EI, one is bound to face distress and depressive symptoms. 

Simply stated, optimism is the foundation of emotional intelligence (EI). Optimism is the ability to remain positive despite obstacles and to believe that obstacles inevitably create the path of change, for it views obstacles or impediments as the steppingstones of achievement and growth. Emotional intelligence (EI) components of self-awareness and recognizing a feeling as it happens, realizing what lies behind a feeling and managing them appropriately, and channeling them in the service of a goal are clear signs of optimistic people who tend to make positive, specific, temporary, and external causal attributions when confronted with setbacks. 

Enhancement of emotional intelligence (EI) alternatively increases hope, which is a positive mental state that motivates people to have control over the evolving events and gaining strength to achieve goals. The ability to have hope in stressful situations is influenced by the cognitive appraisal of the situation and strategic planning of pathways to reach personal goals. Consequently, people with high levels of EI can face challenges, manage conflicts, and fuel themselves by self-encouragement and hope [89]. Some research also indicated that people with high EI remain cheerful and hopeful and even have an optimistic outlook on future life [90]. 

Satisfaction with life also increases with the enhancement of emotional intelligence (EI). As an ability to monitor emotions and use them appropriately, EI guides people through their thinking and actions in creating a satisfying life [91]. Clarity is an important aspect of EI, which aids in the adaptive monitoring and managing of emotions. In other words, people who experience and manage moods clearly may be able to terminate aversive and ruminative processes quickly, in turn, experiencing greater levels of life satisfaction.

Further, with deficits in emotional intelligence (EI), one is bound to face more distress and depressive symptoms. Inversely, higher levels of EI are associated with lower levels of stress and depressive variables. This is because EI has a protective effect regarding reactions to psychological distress. In other words, people’s self-perceptions of their emotional abilities and skills influence their ability to cope successfully with distress and pressures. Moreover, people who monitor and manage emotions clearly may be able to quickly terminate ruminative processes involved in distress and depression. As such, EI intuitively offers a door to mental illness and health, since the ability of individuals to understand and manage their emotional states could be considered as an important indicator of one’s mental functioning. Therefore, it further suggests the useful function of enhanced EI dimensions concerning the promotion of healthy and adaptive mental functioning.

## 5. Conclusions, Implications, and Limitations

This research demonstrated that emotional intelligence (EI) significantly impacts subjective wellness and its counterpart distress. Consequently, it is natural to ask whether emotionally intelligent skills and competencies can be enhanced to improve well-being and buffer distress. The significant results from this study respond positively by suggesting that EI knowledge, skills, and competencies can indeed be enhanced through formal education and structured training. The findings indicate that EI domains are both teachable and learnable and that these can be performed by relatively simple didactic methods over a relatively short period in any sort of setting. The results from this study not only show that the EI enhancing programs can make a significant difference but also support the notion that the EI measures can effectively be used to assess the progress achieved as a result of the EI enhancement programs.

What also needs to be performed in future research projects involving the EI domains and their correlates is to investigate more extensively a variety of pre-intervention and post-intervention instructional and applicational parameters to evaluate the extent to which positive changes have been made as well as maintained over a given time. It is hoped that the results presented in this paper, together with future findings in this specific area involving more variables and populations, will eventually make their way into not only educational institutions but also informal education, social organizations, family environments, and healthcare settings. Leaders, educators, helping professionals, and parents could benefit from learning how best to lead, educate, and care for people so that they become more emotionally intelligent and hopefully create a lasting effect on their overall well-being.

In particular, the understanding of EI could add an important and valuable component to helping professions, wherein the helping personnel could assess their clients’ level of EI, its correlates, and overall well-being and mental functioning. The results of this type of assessment and screening could indicate the need to engage in guidance and counseling sessions designed to strengthen the specific EI deficiencies and thus assist the help-seekers to improve their health and well-being.

Finally, it has to be acknowledged that this particular research project has its limitations. First of all, due to its correlational nature, making causal associations among the study variables should be avoided. Second, the samples used in both the studies and their sizes restrict the generalizability of the results. Third, further studies could be conducted with different cultural samples to replicate these results. Fourth, as indicated in the implications, future studies should include non-academic settings with populations at different developmental stages. Fifth, more EI measures should be explored, and various outcome variables should be included in the analyses. Finally, more appropriate and relevant EI enhancement programs or courses should be designed, so that the research in EI understanding and enhancement can be expanded and its impact on various life domains can be strengthened.

## Figures and Tables

**Table 1:** Sample demographics (Study 1).

Category	Items	Frequency	Percentage
Gender	Male	60	29.7
	Female	141	69.8
	Missing	1	0.5
Age	18	12	5.9
	19	70	34.7
	20	59	29.2
	21	37	18.3
	22	19	9.4
	23	3	1.5
	24	1	0.5
	25	1	0.5
Department	Educational leadership	46	22.8
	Chinese literature	36	17.8
	Clinical psychology	49	24.3
	Life-science	31	15.3
	English literature	40	19.8

*N* = 202.

**Table 2:** Reliability coefficients, means, standard deviations, and correlations (Study 1).

Variables	α	M	SD	1	2	3	4	5	6
1. Emotional Intelligence	0.94	170.20	28.26	---	0.26 **	0.26 **	0.43 **	−0.15 *	−0.28 *
2. Optimism	0.80	20.00	3.96		---	0.51 **	0.39 **	−0.43 **	−0.52 **
3. Satisfaction with Life	0.82	19.08	5.42			---	0.44 **	−0.39 **	−0.52 **
4. Hope	0.74	23.84	3.48				---	−0.06	−0.26 **
5. Psychological Distress	0.82	06.87	4.14					---	0.74 **
6. Depression	0.92	30.87	9.00						---

*N* = 202; ** *p* < 0.01; * *p* < 0.05; two-tailed.

**Table 3:** Emotional management course contents and descriptive statistics (Study 2).

Course Contents	Min	Max	Mean	SD
EM1: Concepts, Definitions, Categories, Functions	3.00	10.00	7.17	1.72
EM2: “The Angry Birds” Movie and Discussion	3.00	10.00	7.04	1.44
EM3: Prominent Figure (Ex: Trump) and Emotional Management	3.00	10.00	6.46	1.77
EM4: Physiology and Emotions	3.00	10.00	7.37	1.76
EM5: Emotions and Cognition	3.00	10.00	7.65	1.64
EM6: EQ Questionnaire and Analysis	3.00	10.00	7.81	1.45
EM7: Personality and Emotions	3.00	10.00	7.11	1.42
EM8: Personality Test and Analysis	3.00	10.00	7.74	1.68
EM9: Emotions in the Context of Family	3.00	10.00	7.67	1.59
EM10: Social Culture and Emotions	3.00	10.00	7.63	1.57
EM11: “Inside Out” Movie and Individual Reflection	3.00	10.00	9.00	1.55
EM12: “Inside Out” Movie, Group Discussion and Oral Report	3.00	10.00	8.15	2.04
EM13: Contemporary Media Influence on Emotions	3.00	10.00	7.39	1.48
EM14: Emotional Management amid Technology Development	3.00	10.00	6.89	1.66
EM15: Exercise on Developing an Emotional Vocabulary	3.00	10.00	6.76	1.72
EM16: Communication Power of Emotions in Interpersonal Relations	3.00	10.00	7.74	1.56
EM17: In-vivo Exercise in Discovering and Expressing Emotions	3.00	10.00	7.28	1.69
EM18: Emotions and Metaphors	3.00	9.00	6.54	1.66
EM19: Exercise on the Relation between Needs, Metaphors, and Emotions	2.00	9.00	6.63	1.72
EM20: Group Exercise on Constructing Emotional Management Pattern	2.00	10.00	7.37	2.03
EM21: Weekly Journaling and Analysis of Emotions	3.00	10.00	7.63	2.03
EM22: Semester Integration Paper (Personalized Emotional Management Pattern)	2.00	10.00	7.02	2.18

*N* = 46. EM: Emotional Intelligence. The numbers 1,2,3…22 are the random sequencing of course contents.

**Table 4:** Reliability coefficients, means, standard deviations, and correlations (Study 2).

**Variables**	**Pre-Course (Time 1)**								
	**α**	**M**	**SD**	**1**	**2**	**3**	**4**	**5**	**6**
1. Emotional Intelligence	0.96	154.39	37.94	---	0.25	0.27	0.61 **	−0.11	−0.28
2. Optimism	0.74	20.22	3.78		---	0.49 **	0.58 **	−0.35 **	−0.41 **
3. Satisfaction with Life	0.91	19.52	6.23			---	0.59 **	−0.38 **	−0.50 **
4. Hope	0.79	27.67	4.22				---	−0.26	−0.47 **
5. Psychological Distress	0.77	07.09	4.15					---	0.74 **
6. Depression	0.89	31.09	8.27						---
	**Post-Course (Time 2)**								
	**α**	**M**	**SD**	**1**	**2**	**3**	**4**	**5**	**6**
1. Emotional Intelligence	0.96	179.37	33.17	---	0.22	0.37 *	0.45 **	−0.19	−0.43 **
2. Optimism	0.76	26.96	1.35		---	0.35 **	0.51 **	−0.14	−0.22
3. Satisfaction with Life	0.92	29.89	2.95			---	0.44 **	−0.29	−0.26
4. Hope	0.79	35.41	2.08				---	−0.10	−0.54 **
5. Psychological Distress	0.78	02.00	1.56					---	0.48 **
6. Depression	0.89	16.54	4.39						---

*N* = 46; ** *p* < 0.01; * *p* < 0.05; two-tailed.

**Table 5:** Results of regression analyses with emotional intelligence as predictor variable (study 1).

Outcome Variables	*F*	*R* ^2^	*t*	*β*	Confidence Intervals (Lower–Upper)
Optimism	14.83 ***	0.06	3.85 **	0.26	0.018~0.057
Satisfaction with Life	14.86 ***	0.07	3.85 **	0.26	0.025~0.078
Hope	46.28 ***	0.18	6.80 **	0.43	0.056~0.102
Psychological Distress	4.32 *	0.02	−2.08 *	−0.14	−0.043~−0.001
Depression	17.26 ***	0.08	−4.15 ***	−0.28	−0.135~−0.048

*N* = 202; * p < 0.05; ** p < 0.01; *** p < 0.001.

**Table 6:** Results of paired-samples (repeated measures) *t*-test (study 2).

Variables	Mean Difference	Confidence Interval		*t* (*df* = 45)	η2
		Lower	Upper		
Emotional Intelligence	24.98	−30.77	−19.17	8.67 *	0.63
Optimism	6.74	−7.74	−5.73	13.51 *	0.80
Satisfaction with Life	10.37	−11.60	−9.13	16.97 *	0.86
Hope	7.74	−8.60	−6.87	17.99 *	0.88
Psychological Distress	5.08	4.23	5.94	12.03 *	0.76
Depression	14.54	13.03	16.05	19.40 *	0.89

*N* = 46; * *p* < 0.001; two-tailed.

**Table 7:** Results of regression analyses with emotional intelligence as predictor variable (study 2).

Time	Outcome Variables	*F*	*R* ^2^	*t*	*β*	Confidence Intervals (Lower–Upper)
Pre-Course	Optimism	2.92	0.06	1.70	0.25	−0.004~0.054
(Time 1)	Satisfaction with Life	3.41	0.07	1.85	0.27	−0.004~0.092
	Hope	26.08 **	0.37	5.11 ***	0.61	0.041~0.095
	Psychological Distress	0.57	0.01	−0.76	−0.11	−0.045~0.021
	Depression	3.83	0.08	−1.96	−0.28	−0.126~0.002
Post-Course	Optimism	2.17	0.05	1.47	0.22	−0.003~0.021
(Time 2)	Satisfaction with Life	6.80 *	0.13	2.61 *	0.37	0.007~0.058
	Hope	10.82 **	0.20	3.29 **	0.44	0.011~0.045
	Psychological Distress	1.69	0.04	−1.30	−0.19	−0.023~0.005
	Depression	9.96 **	0.19	−3.16 **	−0.43	−0.092~-0.020

*N* = 46; * p < 0.05; ** p < 0.01; *** p < 0.001.

## Data Availability

Data for the current study are available upon written request.
